# Paper-based LRET sensor for the detection of total heavy rare-earth ions

**DOI:** 10.3389/fchem.2022.1028441

**Published:** 2022-10-04

**Authors:** Qiang Chen, Keren Tang, Dengwang Luo, Luodan Han, ChunXiao Yu, Yiping Shen, Qi Lin, Yiting Chen, Chunyan Li, Jinghua Chen, Jianming Lan

**Affiliations:** ^1^ College of Materials and Chemical Engineering, MinJiang University, Fuzhou, China; ^2^ The School of Pharmacy, Fujian Medical University, Fuzhou, China; ^3^ CAS Key Laboratory for Biological Effects of Nanomaterials and Nanosafety, National Center for Nanoscience and Technology, Chinese Academy of Sciences, Beijing, China

**Keywords:** luminescence resonance energy transfer, DNAzyme, upconversion nanoparticles, AuNPs, rare-earth ions

## Abstract

Based on the mechanism of luminescence resonance energy transfer (LRET) and using a special single strand DNA as the recognition element, a portable paper-based sensor for the accurate detection of total heavy rare-earth ions (mainly Gd^3+^, Tb^3+^ and Dy^3+^) concentration was proposed. The RNA cleaving-DNAzyme should recognize rare-earth ions to cleave RNA on DNA duplexes linking UCNPs and AuNPs, causing UCNPs and AuNPs to approach each other, inducing LRET, which attenuated the green upconversion luminescence (UCL) triggered by the 980 nm laser. UCL was captured by a charge-coupled device (CCD) image sensor and processed with the red-green-blue (RGB) image to quantitatively analyze heavy rare-earth ions in the samples. In the range of 5–50 μmol·L-1, the sensor has good sensitivity, with the limit of detection of 1.26 μmol L^−1^.

## Introduction

Rare-earth elements (REEs) are characterized by unique optical (Cho and Chen, 2020), magnetic (He et al., 2022), and catalytic properties (Kim et al., 2021) and are known as “industrial vitamins.” These materials are used widely in various fields such as chemical engineering and metallurgy, and also are used for the fabrication of specific materials. The large-scale exploitation and use of REEs have resulted in serious damage to the geo-environment, aquatic plants and animals, and human life (Adeel et al., 2019). The REES presented in the environment enter the human body through the food chain and negatively affect human health (Huo et al., 2017). Therefore, the detection of rare earth elements is an urgent problem to be solved. Presently, the detection techniques of REES are primarily realized by using such as high-performance liquid chromatography (HPLC) (Pedreira et al., 2003), atomic absorption spectroscopy (AAS) (Jarvis et al., 1992), and inductively coupled plasma atomic emission spectroscopy (ICP-AES) (Naumova et al., 2015), etc. These detection methods, however, all require the use of complex preprocessing or massive and costly instruments, making rapid detection and outdoor application difficult to achieve. Moreover, due to the highly similar chemical structures and properties of different REEs (Stamberga et al., 2020), those optical and electrochemical sensors fabricated from small-molecule chelators typically show low selectivity and sensitivity (Kolarik et al., 2008; Vargas-Zúñiga and Sessler, 2012).

Recently, studies on DNA-based biosensors to detect metal ions have attracted great attention. Researchers have screened a large number of DNA sequences *in vitro* to locate DNAzymes that give specific responses to different metal ions for the fabrication of biosensors. Among them, DNAzymes for Pb^2+^ detection have been widely used (Wen et al., 2011; Liu et al., 2012), and colloidal gold (AuNP)-based DNAzymes also have been used for the detection of Zn^2+^ and Cu^2+^ presented in living cells and tissues (Li et al., 2015). Furthermore, the use of DNAzymes for the detection of rare-earth ions has rarely been reported (Huang et al., 2014a; Huang et al., 2014b). DNAzymes are DNA-based biocatalysts, which exhibit catalytic activity in the presence of metal ions that function as cofactors (Navani and Li, 2006). Recent work indicates that RNA-cleaving DNAzymes can be used as powerful metal ion sensing platforms because they exhibit rapid cleavage rate and strong binding affinity toward metals (Xiao at., 2007; Lu et al., 2011). After years of research, Liu et al. reported five RNA-cleaving DNAzymes that had been used to develop sensor arrays for the identification of different REEs. Among them, a novel DNAzyme named Gd2b could be used to effectively distinguish Ln^3+^ from other metal ions. It was also capable of differentiating between light Ln^3+^ ions and heavy Ln^3+^ ions (Huang et al., 2016).

Moreover, due to its simple operation, high sensitivity and fast response characteristics, fluorimetry has unique advantages for the detection of metal ions. In terms of fluorescence technology, it is more advantageous to use rare-earth doped upconversion nanoparticles (UCNPs) compared with traditional “down-conversion” fluorescent materials, such as organic dyes (Li Z. J. et al., 2021), quantum dots (Li W. T. et al., 2021), and carbon nano-materials (Rashid et al., 2021). The UCNPs are characterized by narrow emission bands and high photostability, and no autofluorescence interference. To date, many different types of upconversion luminescence (UCL) probes based on the inner filter effect (IFE) or luminescence resonance energy transfer (LRET) have been used to detect metal ions that are abundantly present in complex systems (Chen M. et al., 2018; Li et al., 2018).

In view of the fact that China is rich in rare-earth minerals, especially the reserves of rare-earth minerals are rich in heavy rare-earth ions mainly represented by gadolinium (Gd), terbium (Tb), and dysprosium (Dy). However, in the process of mining and processing, a large amount of heavy rare-earth ions will be discharged. The characteristic pollutant of rare-earth ions is usually wastewater, which seriously pollutes the water environment. Guided by these results, we proposed a disposable paper-based LRET sensor for the detection of the total quantity of chemically similar heavy rare-earth ions (mainly Gd^3+^, Tb^3+^ and Dy^3+^) presented in water samples. Using UCNPs and AuNPs combined with DNA technology, the sensor can be used as an outdoor portable detector for environmental monitoring by virtue of sensitive LRET and specific recognition ability of DNAzyme.

## Experimental

### Instruments and reagents

We used Perkin Elmer Spectrum 2000 FT-IR spectrometer (Perkin Elmer, Waltham, MA, USA), UV-2600 UV spectrophotometer (Shimadzu, Kyoto, Japan), JEM-2100 EX transmission electron microscope (TEM; JEOL, Tokyo, Japan), MiniFlex 600 X-ray powder diffractometer (XRD; Rigaku, Tokyo, Japan), SH-4000M scanning electron microscope (SEM; Hirox, Tokyo, Japan), Cary Eclipse fluorescence spectrophotometer (Agilent, Santa Clara, CA, USA), Litesizer 500 nanoparticle size/zeta potential analyzer (Anton Paar, Graz, Austria), Cannon 60-D digital camera (Canon, Tokyo, Japan), fluorescence imaging system (Mshot Optoelectronics Technology, Guangzhou, China), and MDL-III 980 nm laser (Changchun Optics Technology, Changchun, China) to conduct the experiments.

Genetic fragments were synthesized by Sangon Biotech (Shanghai, China) and purified using the HPLC technique. The following sequences prepared have been presented as substrate (5′-ACGAGTCACTATrAGGAAGATGGC-3′) and DNAzyme (5′-SH-TTTTTTTTTTCGCCATCTTGACGCATA TCGTTTTCGAT AGCACGTGTTAGTGACTCGTGAC-NH_2_-3′). The reagents used include oleic acid (OA), octadecene (ODE), polyethyleneimine (PEI), and chloroauric acid. All the reagents were obtained from Shanghai Aladdin Bio-Chem Technology (Shanghai, China). Stearates of REEs (Y, Yb, and Er) were synthesized by our group. Other reagents used were analytical grade and purchased from the Sinopharm Group (Shanghai, China). Water (conductivity, 0.0548 μS cm^−1^) was purified by using the Milli-Q system (Burlington, MA, USA).

### PEI modification of UCNPs

NaYF_4_: Yb, Er UCNPs were synthesized by a thermal decomposition reaction, which was introduced in detail in Supplementary Section S1. OA ligands present on the surface of UCNPs were replaced by NOBF_4_ following the process of ligand exchange (Liang et al., 2017). This was achieved by mixing 10 ml of OA-coated nanoparticles (about 10 mg mL^−1^) dispersed in hexane with 20 ml of NOBF_4_ (0.01 M) in *N*,*N′*-dimethylformamide (DMF). After stirring at room temperature for 2 h, the bottom phase was purified using toluene and hexane (1:1, v/v), and then centrifuged. The obtained ligand-free UCNPs were washed twice with DMF and dispersed in 5 ml DMF, which was then mixed with 15 ml of DMF solution containing 1.0 g PEI. After stirring overnight, the mixture was washed with deionized water, and then vacuum-dried at 60°C.

### Preparation of AuNPs-DNAzyme

The synthesis of AuNPs is described in Supplementary Section S2. The DNAzyme was modified onto the AuNP surface by Au-S bond. In short, equal amounts of DNAzyme and substrate were mixed in 50 mM 4-(2-Hydroxyethyl)piperazine-1-ethanesulfonic acid (HEPES) buffer (300 mM NaCl, pH 7.0). After heating at 90°C for 5 min, the mixture was cooled to room temperature. Then mixing with the sodium citrate-encapsulated AuNPs solution at a molar ratio of 200:1, the mixture was then frozen at −20°C for 2 h (Liu and Liu, 2017, 2019). A 0.3 M of sodium chloride solution was added to thaw the mixture. After centrifugation, the resulting AuNPs-DNAzyme was redispersed in HEPES buffer and stored at 4°C.

### Oxidation of filter paper

A filter paper (Whatman grade 1) was cut into 3 mm-diameter pieces with the puncher and placed in a conical flask. NaOH solution (14%) was added to activate the filter paper pieces. The process of activation was performed over 24 h. After the pieces were rinsed with distilled water, the oxidizing agent (26 mM NaIO_4_, 47 mM LiCl, pH 2.0) was added to oxidize the pieces for 2 days at 35°C. Finally, the pieces were rinsed with distilled water, and dried at 40°C (He et al., 2016).

### Anchoring of UCNPs and AuNPs-DNAzyme

PEI-UCNPs solution (0.1 mg mL^−1^) was prepared in HEPES buffer (100 mM, 200 mM NaBH_3_CN, pH 7.2). The solution (3 μL) was dropped freely onto a piece of the cut filter paper. After reacting at room temperature for 1 h, all unfixed PEI-UCNPs were washed off by using 0.2% Tween 20 and ultrapure water. After drying, 3 μL of 50% glutaraldehyde was added dropwise to each piece of filter paper, and then aliquots (3 μL) of AuNPs-DNAzyme at different concentrations (0.0, 2.0, 4.0, 6.0 8.0 nM) were dropped dropwise onto 5 pieces of cut filter paper, respectively, and reacted at room temperature for 1 h and washed three times with ultrapure water.

### Rare-earth ions detection in water samples

Aliquots (3 μL) of tap water samples with total heavy rare-earth ions (Equal ratios of Gd^3+^, Tb^3+^, and Dy^3+^ ions) at different concentrations (10, 15, and 50 μmol L^−1^) were dropped freely onto a piece of cut filter paper kit. After reacting at room temperature for 2 h, the UCL was generated by exciting by 980 nm laser (0.5 W cm^−2^) in a dark box. The green luminescence image was captured using a CCD image sensor and processed with RGB image system software. The standard curve was plot by UCL quenching rate versus rare-earth ion concentrations.

## Results and discussion

### Principle of heavy rare-earth ions detection

**SCHEME 1:**
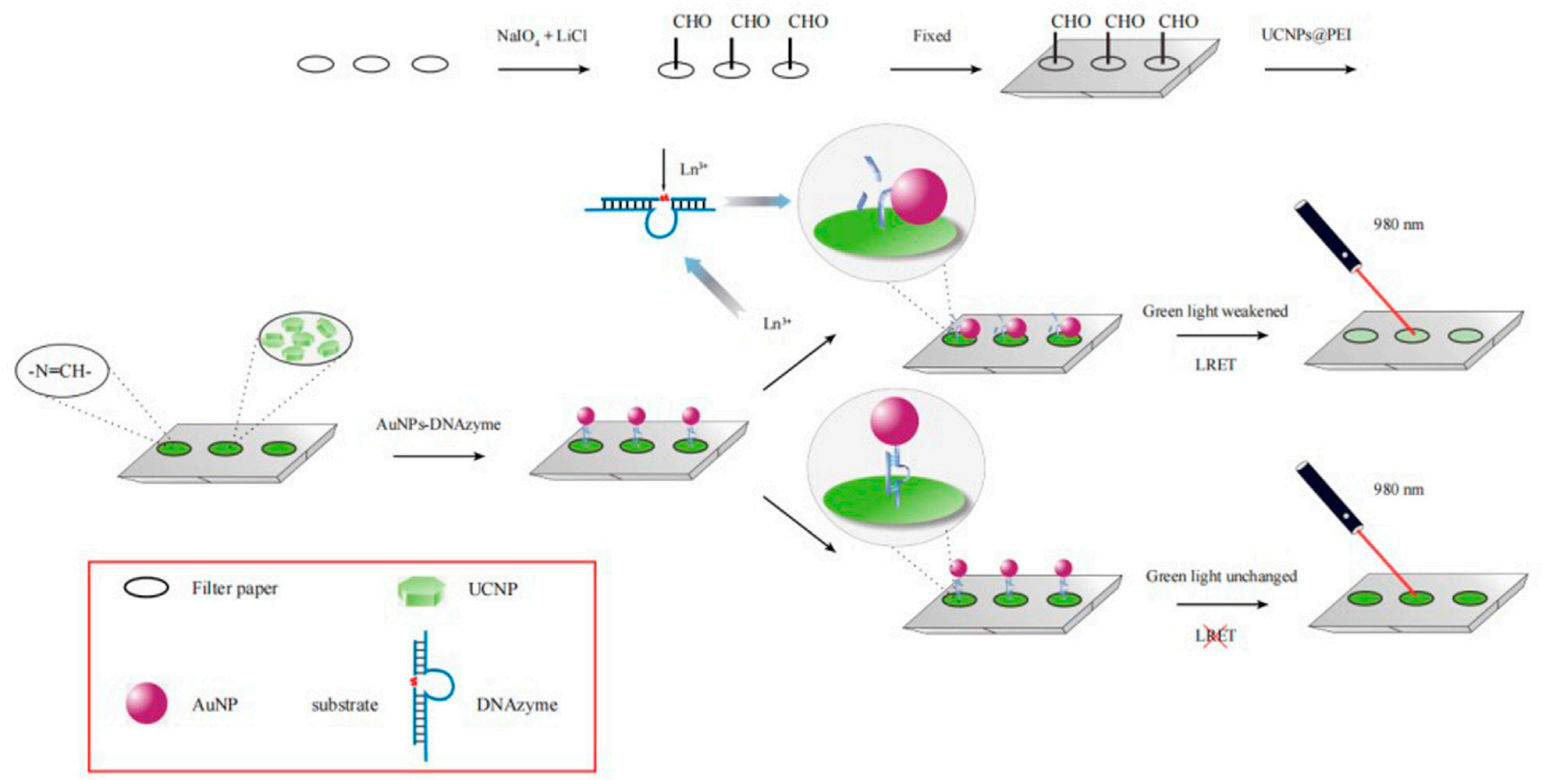
Mechanism of detecting rare-earth ions based on LRET.

The detection mechanism is shown in Scheme 1. PEI- UCNPs were added to the surface of filter paper oxidized with sodium periodate. The amino groups on PEI reacted with the aldehyde groups on the surface of the oxidized filter paper by a Schiff-base reaction, thereby immobilizing the UCNPs on the surface of the oxidized filter paper. The substrate modified with RNA linkage (ribo-adenosine, rA) in the middle and the DNAzyme modified with -SH group at the 3′ end (substrate:enzyme = 1:1) were hybridized to form duplexes, which were linked to AuNPs through Au-SH bonds. When AuNPs-DNAzyme was added to the surface of the filter paper, Amino-modified DNA was cross-linked with amino groups on the surface of PEI-UCNPs through glutaraldehyde cross-linking. The formation of stable and rigid structure of DNA duplexes would keep AuNPs away from UCNPs, without causing LRET. In the presence of rare-earth ions, bimetallic clusters ([Ln_2_(OH)_2_]^4+^) formed readily with water molecules. These clusters exhibited a strong affinity toward the phosphate groups present in DNA. They could be used to specifically recognize the hammerhead motif formed by long strands DNAzyme, thus acting on the cleavage site of rA (Supplementary Figure S1A). Under these conditions, the bimetallic cluster [Ln_2_(OH)_2_]^4+^ acted as a nucleophilic reagent to neutralize the charge on the phosphate group. The cluster also activated the 2′-OH unit, which functioned as a nucleophile and attacked the phosphorus atom present in the phosphate group. It also induced the cleavage of the phosphate-diester bond, resulting in hydrolysis of RNA linkage (Komiyama et al., 1999). As the hydrogen bond broke, the separated short DNA chain became unstable and detached immediately. The long-chain enzyme bent, shortening the distance between the UCNPs and AuNPs. Upon excitation by the 980 nm laser, the LRET occurred between the two. The UCL of the UCNPs was quenched, and the intensity of green luminescence weakened (Supplementary Figure S1B). Through RGB analysis, the relationship between the quenching rate of UCL and the concentrations of heavy rare-earth ions was determined, thereby realizing high-sensitivity detection of heavy rare-earth ions.

### Characterization of UCNPs

**FIGURE 1:**
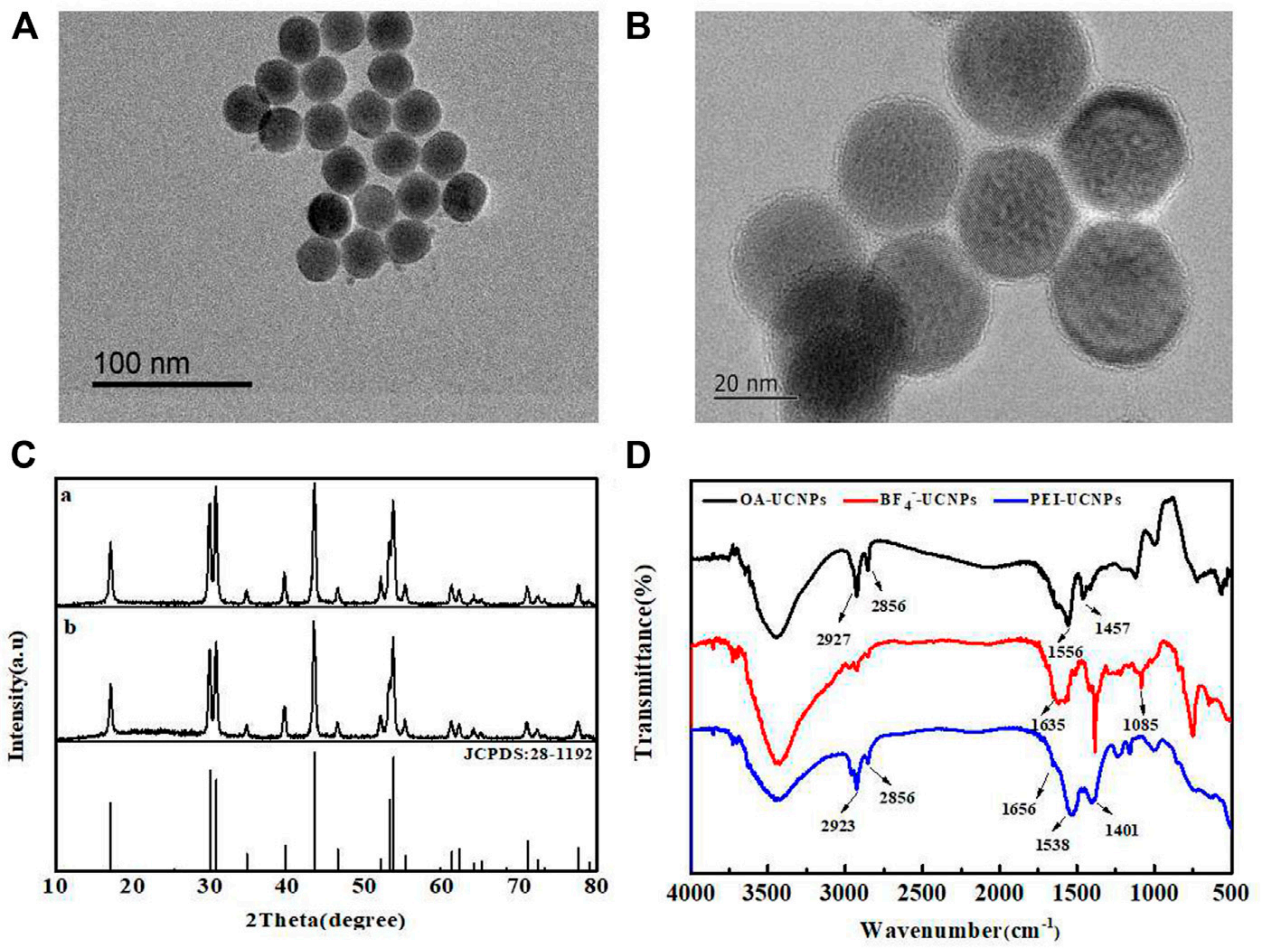
**(A)** TEM image of OA-UCNPs. **(B)** TEM image of PEI-UCNPs. **(C)** XRD patterns recorded for OA-UCNPs **(a)** and PEI-UCNPs **(b)**. **(D)** FT-IR spectra recorded for OA-UCNPs, BF_4_^−^-UCNPs and PEI-UCNPs.

Figure 1A presents the TEM image recorded for OA-UCNPs. These particles were uniform in size and well-dispersed. The average particle size was approximately 25 nm, and the good dispersion of PEI-UCNPs was observed from Figure 1B. Figure 1C presented the XRD patterns. A comparison with the standard spectrum of NaYF_4_ (JCPDS: 28-1192) revealed that the synthesized UCNPs were of the hexagonal (β) phase. Figure 1D presents the FT-IR spectra recorded for the PEI-UCNPs. The characteristic peak at 3436 cm^−1^ corresponded to the stretching vibration peaks of O-H or N-H. The peaks at 2,923 cm^−1^ and 2,856 cm^−1^ corresponded to the asymmetric and symmetric stretching vibration of -CH_2_ in PEI, respectively. 1638 cm^−1^ and 1538 cm^−1^ corresponded to the bending peaks of N-H groups present in PEI, and the peak at 1164 cm^−1^ corresponded to the C-N stretching vibration. Moreover, as shown in Supplementary Figure S2, the Zeta potential of UCNPs after being modified with positively charged PEI was significantly increased. These results revealed that the PEI containing amino groups had been successfully assembled onto the surface of UCNPs.

### Characterization of AuNPs-NDAzyme

**FIGURE 2:**
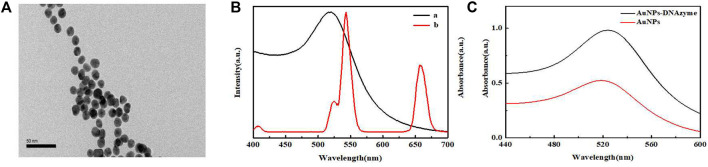
**(A)** TEM image of AuNPs. **(B)** UV-vis absorption spectrum recorded for AuNPs **(a)** and the UCL spectrum recorded for UCNPs **(b)**. **(C)** UV-vis absorption spectra recorded for AuNPs and AuNPs- DNAzyme.

Figure 2A presents the TEM image recorded for the AuNPs. The nanoparticles were well-dispersed and exhibited good uniform morphology, with an average particle size of about 13 nm. Figure 2 presents the UV-vis absorption spectrum recorded for the AuNPs and the UCL emission spectrum recorded for the UCNPs. The maximum absorption wavelength of AuNPs was 518 nm, and the concentration was determined to be approximately 4 nM according to the Beer-Lambert’s Law, with the extinction coefficient (ε) of 2.7 × 10^8^ L mol^−1^·cm^−1^. In this experiment, UCNPs acted as energy donors, and the AuNPs functioned as energy receptors. Analysis of the spectra revealed a high degree of overlapping at 540 nm, indicating that efficient LRET could be generated between UCNPs and AuNPs. Figure 2C presents the UV-vis absorption spectra recorded for the unmodified AuNPs and AuNPs-DNAzyme. A 5 nm redshift was observed in the maximum absorption of the AuNPs-DNAzyme, indicating that DNAzyme-SH had successfully bound to the surface of AuNPs through an Au-S bond. Furthermore, the dynamic light scattering (DLS) analysis of AuNPs also confirmed that DNAzyme had be well modified on the surface of AuNPs, as shown in Supplementary Figure S3. It was further proved in salt-tolerance test of AuNPs-DNAzyme that DNAzyme could prevent the aggregation of AuPNs, as shown in Supplementary Figure S4.

### Characterization of filter paper

**FIGURE 3:**
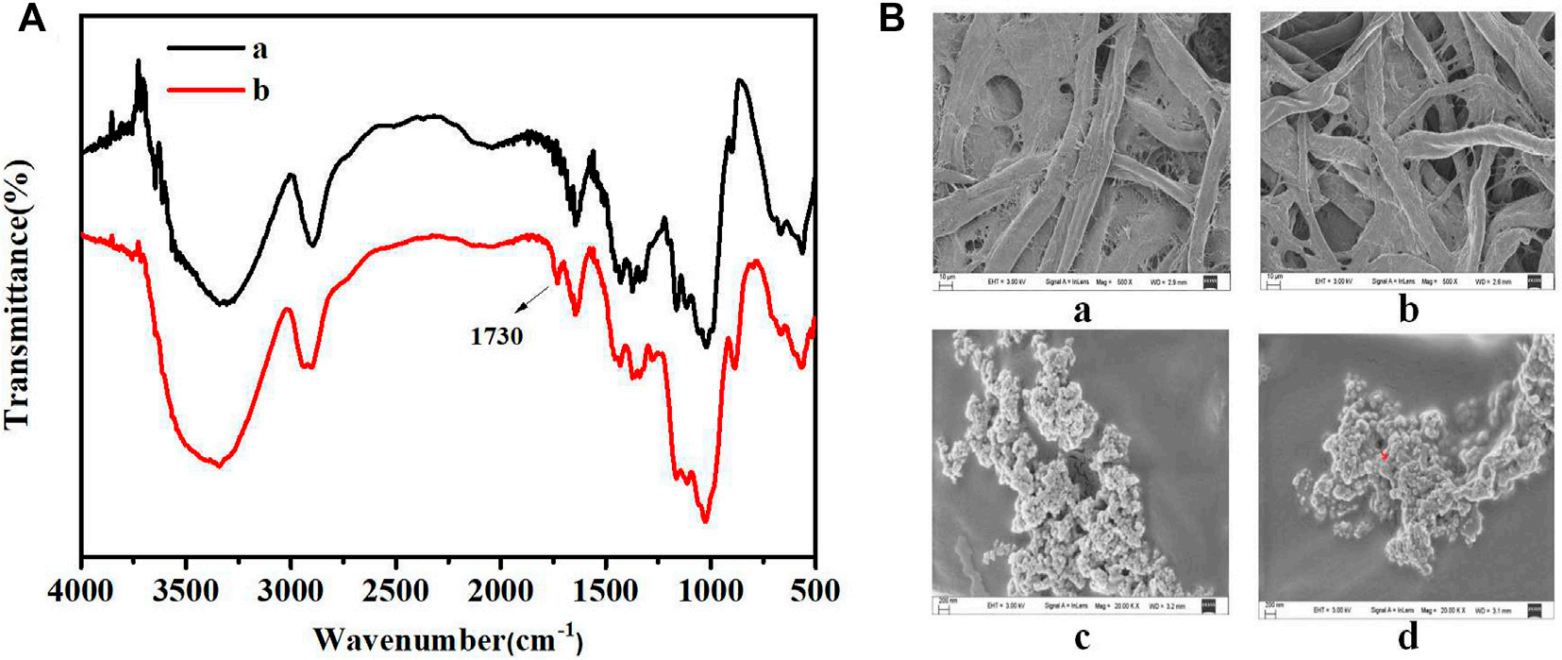
**(A)** FT-IR spectra recorded for filter paper (a) and oxidized filter paper (b). **(B)** SEM images of filter paper: untreated filter paper **(a)**, oxidized filter paper **(b)**, oxidized filter paper immobilized with PEI-UCNPs **(c)**, oxidized filter paper immobilized with PEI-UCNPs and AuNPs-DNAzyme **(d)**.

Figure 3A presents the FT-IR spectra recorded for the filter paper and oxidized filter paper. The primary constituent of filter paper was cellulose containing a large number of o-hydroxyl groups in its structure. With the joint action of lithium chloride and sodium periodate, the o-hydroxyl groups were oxidized to o-dialdehydes. The spectrum of oxidized filter paper reveals that 1730 cm^−1^ corresponds to the aldehyde (-CHO) stretching vibration, confirming the oxidation of o-diols to o-dialdehydes. These results agree well with previously reported results (Chen X. S. et al., 2018).

Figure 3B presents the SEM images recorded for the filter paper. As seen in Figures 3Ba,b, oxidation did not damage the microscopic structure and pores of the paper matrix. Analysis of Figure 3Bc revealed that the PEI-UCNPs could be successfully fixed and well-dispersed on the cellulose units of the oxidized filter paper matrix. Figure 3Bd revealed a uniform dispersion of AuNPs-DNAzyme on the cellulose units modified with PEI-UCNPs.

### Anchoring time and stability of the PEI-UCNPs on the surface of the filter paper

**FIGURE 4:**
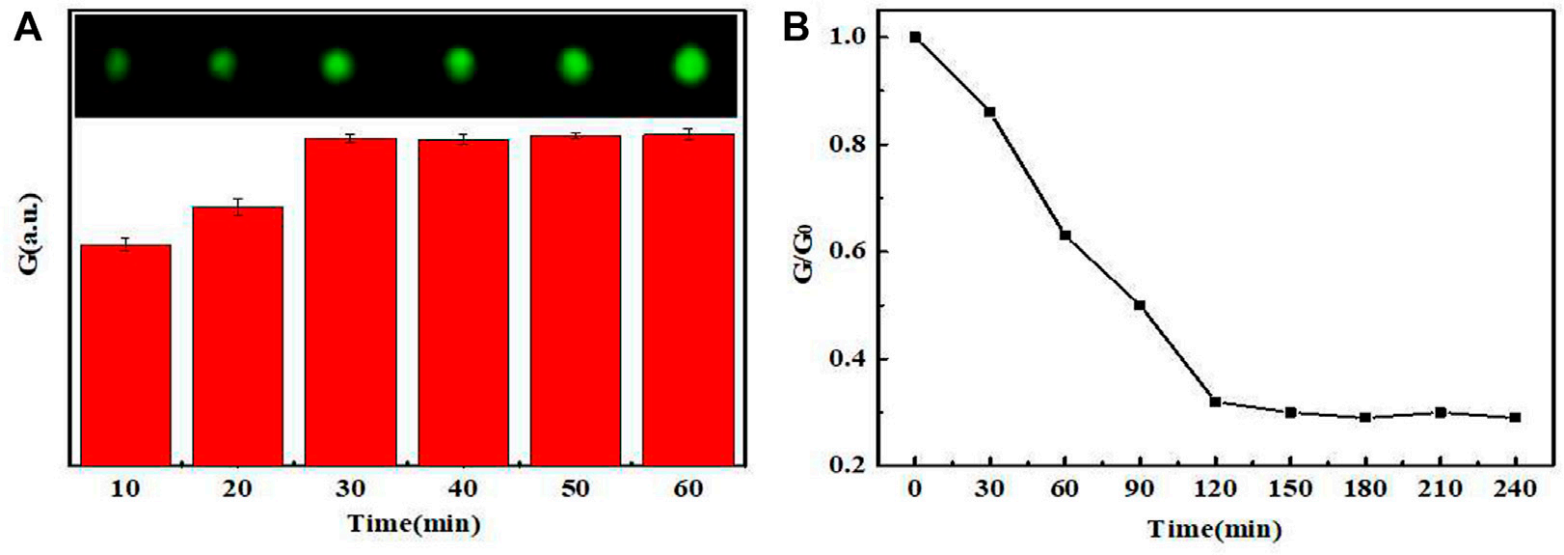
**(A)** The UCL intensity graph and image spots of PEI-UCNPs at different fixed times. **(B)** The UCL intensity varied with different times.

When the dosage of PEI-UCNPs solution was 0.1 mg mL^−1^, CCD was used to capture the UCL image. The color picker in the drawing software was used to collect the UCL spot, and the RGB value of the spot was determined using the color editor. As shown in Figure 4, the UCL intensity of the spots varied with the time of aldimine condensation reaction. When the reaction time reached 60 min, the UCL spot was the brightest and completely uniform. Furthermore, the stability of the UCL intensity verified that the anchoring effect of PEI-UCNPs on the oxidized filter paper was better than that on the filter paper, and it was further verified that the stability of the PEI-UCNPs immobilized on the oxidized filter paper after soaking treatment was better than that of after ultrasonic treatment, as shown in Supplementary Figure S5. The results of Supplementary Figure S6 showed that the intensity of UCL remained unchanged within a week, indicating that the PEI-UCNPs fixed on the oxidized filter paper surface were very stable.

### Optimization of reaction time

The steric hindrance exerted by DNAzyme made it difficult for AuNPs and UCNPs to approach each other. Without adding heavy rare-earth ions, the results of Supplementary Figure S7 showed that the UCL was hardly quenched with the increasing concentration of AuNPs-DNAzyme, indicating that the design of the sensing mechanism is feasible. In the presence of heavy rare-earth ions, the concentration of AuNPs-DNAzyme affected the UCL quenching rate of UCNPs on filter paper. As shown in Supplementary Figure S8, when the concentrations of PEI-UCNPs and the rare earth ion were 0.1 mg mL^−1^ and 10 μM, respectively, 6 nM AuNPs-DNAzyme could saturate the quenching of UCL.

After determining the above dosages of UCNPs, AuNPs-DNAzyme and rare-earth ions, we further studied the relationship between the quenching rate of UCL and the reaction time. As shown in Figure 4B, the rare-earth ions continued to cut DNAzyme until 120 min. Subsequently, the cutting activity gradually decreased, and the UCL intensity decreased accordingly. Therefore, 120 min was taken as the optimal reaction time.

### Selectivity of sensor

**FIGURE 5:**
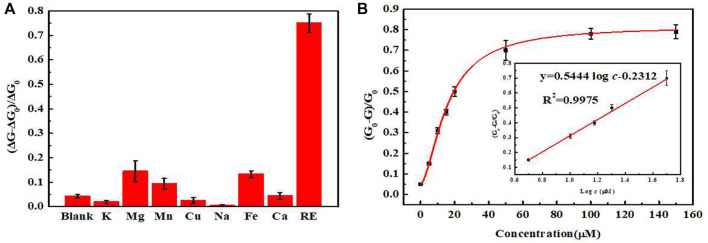
**(A)** Selectivity of the sensor toward different metal ions (Target: a mixture of Gd^3+^, Tb^3+^ and Dy^3+^ ions in equal proportions). **(B)** Relationship between the concentrations of rare-earth ions and quenching rate. (Insert: the standard curve of this sensor).

In order to evaluate the specificity of the sensor to rare-earth ions, the standard concentration of rare-earth ions in the tested samples was formulated as 10 μmol L^−1^, and the concentrations of the interfering ions such as K^+^, Na^+^, Mg^2+^, Mn^2+^, Ca^2+^, Fe^3+^, Y^3+^, La^3+^, and Lu^3+^ were standardized to 100 μmol L^−1^ as control group. As shown in Figure 5, all the interfering ions (in which Y^3+^, La^3+^, and Lu^3+^ ions were represented as light rare-earth ions) exerted little influence on the UCL intensity of the sensor, indicating no interference on the measurement of heavy rare-earth ions. Thus, the high selectivity of designed sensor ensured that it could be used for the detection of the total amount of heavy rare-earth ions in complex environment.

### Sensitivity of sensor

We tested standard mixtures of rare-earth ions of different concentrations using the optimized experimental conditions. The UCL intensity was measured and plotted to generate a working curve. In the range of 5–50 μmol L^−1^, the UCL intensity and the concentrations of rare-earth ions in the standard mixtures exhibited a good linearity. The fitted regression equation was y=0.5444⁡log⁡c−0.2312 , the correlation coefficient was 0.9967, with the limit of detection (LOD) of 1.26 μmol L^−1^.

In addition, we reviewed recent studies on the detection of different rare-earth ions, summarized in Table 1. We compared the detection mathod, principle, target, linear range and LOD of our sensor with those reported so far. At present, most of the studies used colorimetriy and fluorimetry, which had better sensitivity. In terms of the selecting detection objects of rare-earth ions, most of them were light rare-earth ions, such as Ce^3+^, La^3+^, Eu^3+^, etc., while the detection of heavy rare-earth ions was less. For the detection mechanism, most of the coordination strategies were used to realize the specific recognition of the target, but it lacked good selectivity in distinguishing numerous rare-earth ions. Our sensor used RNA-cleaving DNAzyme to achieve specific recognition of heavy rare-earth ions (mainly including Gd^3+^, Tb^3+^ and Dy^3+^), with better selectivity. Moreover, our sensor used the UCL of UCNPs as the signal source, which had high sensitivity and anti-interference ability, and could realize portable operation by paper matrix. At present, the biggest disadvantage of our sensor was that the incubation time was still relatively long. Therefore, it is necessary to achieve rapid on-site detection by further shortening the incubation time.

**TABLE 1:** Comparison of the different methods for the detection of rare-earth ions.

Method		Principle of analysis	Target	Linear range (M)	LOD (M)	Ref.
1	Colorimetry	Conjugate adsorption	Ce^3+^	1.4 × 10^−8^−7.0 × 10^−7^	2.35 × 10^−9^	Kubra et al. (2021)
2	Colorimetry	Mimetic peroxidase	Ce^3+^	1.0 × 10^−8^−1.6 × 10^−7^	2.2 × 10^−9^	Deng et al. (2019)
3	Fluorimetry	Coordination	La^3+^	0−6.0 × 10^−6^	4.5 × 10^−8^	Wang et al. (2019)
			Sm^3+^	0−1.0 × 10^−5^	2.9 × 10^−5^	
4	Fluorimetry	Aggregation-induced emission	Ce^3+^	0−1.8 × 10^−5^	2.27 × 10^−6^	Wang et al. (2018)
5	Phosphorimetry	Coordination	Gd^3+^	4.0 × 10^−5^−2.2 × 10^−4^	1.0 × 10^−5^	Li et al. (2022)
6	Fluorimetry	Aggregation effect	Eu^3+^	0−5.2 × 10^−4^	1.14 × 10^−6^	Che et al. (2020)
7	Fluorimetry	Coordination	Er^3+^	3.0 × 10^−9^−1.0 × 10^−7^	0.28 × 10^−9^	Dai et al. (2019)
8	Fluorimetry	LRET based on DNAzyme	Gd^3+^, Tb^3+^ and Dy^3+^	5.0 × 10^−6^−5.0 × 10^−5^	1.26 × 10^−6^	This work

### Sample analysis

Tap water samples were collected and filtered through 0.22 µm aqueous phase filter membranes. Sample recovery was then determined following the standard addition method. As shown in Table 2.The results revealed that the method could be used to realize a recovery in the range of 90.5–109.2%, the relative standard deviation (RSD) was in the range of 2.23–5.81%. Therefore, this assay is promising for the detection of total heavy rare-earth ion content in actual water samples.

**TABLE 2:** The test of sample spike recovery (n = 5).

Sample	Added (μmol·L^−1^)	Determined (μmol·L^−1^)	Recovery (%)	RSD (%)
1	10	9.05	90.5	2.23
2	15	16.38	109.2	5.81
3	50	49.55	99.1	4.87

## Conclusion

We fabricated an AuNPs-DNAzyme-labeled filter paper as portable support to measure the total concentration of the chemically similar heavy rare-earth ions, mainly including Gd^3+^, Tb^3+^ and Dy^3+^. Due to the merits of UCNPs and RNA-cleaving DNAzyme, the quantitative analysis of total heavy rare-earth ions can be realized through efficient CCD signal acquisition and rapid RGB analysis. It provides a new sensor in the field of online monitoring of heavy REEs present in wastewater generated by the rare-earth industry. It is expected that real-time terminal data analysis will be realized through smartphones in the future.

## Data Availability

The original contributions presented in the study are included in the article/Supplementary Material, further inquiries can be directed to the corresponding authors.
