# Supplementary material for: Paper-based LRET sensor for the detection of total heavy rare-earth ions
Source: Front Chem. 2022 Oct 4;10:1028441. doi: 10.3389/fchem.2022.1028441 (PMC9577015; doi:10.3389/fchem.2022.1028441)
Supplement: Supplementary file 1 [file DataSheet1.ZIP › Supplementary Material/Supplementary Material.docx]

Supplementary Material

# Synthesis of UCNPs

We synthesized NaYF_4_:Yb,Er UCNPs following established procedures previously published by our group (Liu et al., 2011). OA (12 mL), ODE (8 mL), prepared stearates of REEs (Y, Yb, and Er; 8 mmol), and NaF (28 mmol) were taken in a clean three-neck flask. The mixture was dewatered and degassed by refluxing at 145 °C for 30 min. A clear liquid was obtained. The reaction temperature was quickly rose to 315 °C for 45 min. After this complete reaction, the flask was cooled to room temperature, and the mixture was centrifuged. After discarding the supernatant, the precipitate was collected and washed with cyclohexane, ethanol, and deionized water for several times. The obtained product was vacuum-dried at 60 °C for later use.

# Synthesis of AuNPs

We synthesized AuNPs following previously reported procedures (Lee et al., 2020). Purified water (49.75 mL) was added into a three-neck flask (100 mL). Following this step, 250 μL of 2% chloroauric acid was added to the flask. The resulting solution was heated to boiling. Subsequently, after adding 3 mL of 1% sodium citrate solution, the mixture was stirred continuously for 15 min until it turned purplish-red, and then cooled and stored at 4 °C.

# The dissociation process of DNA duplexes

As shown in Supplementary Figure S1A, the DNA duplexes were formed by the hybridization of a short strand DNA sequence (Substrate: (5′-ACGAGTCACTATrAGGAAGATGGC-3′) and a long strand DNA sequence (RNA cleaving-DNAzyme: 5′-SH-TTTTTTTTTTCGCCATCTTGACGCATA TCGTTTTC GATAGCACGTGTTAGTGACTCGTGAC-NH2-3′). The substrate is flanked by two short base paired duplexes, holding the single ribo-adenosine (rA) to its proximity. This RNA linkage is the chosen cleavage site since it has much lower stability than the rest DNA linkages (Borggrafe et al., 2022). The bimetallic cluster [Ln_2_(OH)_2_]^4+^ be used to specifically recognize the hammerhead motif formed by long-chain DNAzyme, thus acting at the site of rA, resulting in hydrolysis of RNA linkage.

When the substrate had been cut at the site of rA, the stable and rigid structure of DNA duplexes would be disintegrated, resulting in LRET between AuNPs and UCNPs due to the proximity of the distance. as shown in Supplementary Figure S1B.

**
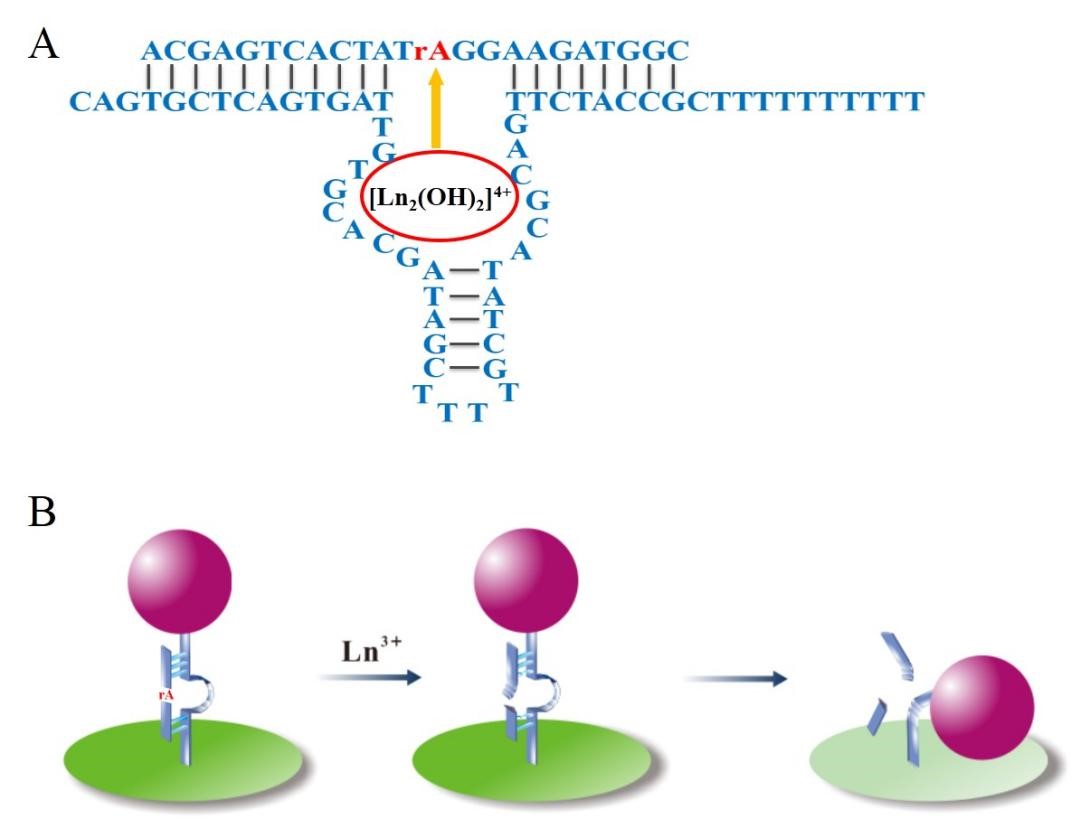
**

**Supplementary Figure 1.** (A) The structure of DNA duplex and the action site of rare-earth ion. (B) Change in the distance between AuNP and filter paper matrix before and after the dissociation of DNA duplex.

# Zeta potentials of UCNPs

The characterization of Zeta potentials of UCNPs showed that the potential became significantly positive after removing oleic acid with NOBF_4_, and the potential became further positive after modifying the positively charged PEI, as shown in Supplementary Figure S2.

#
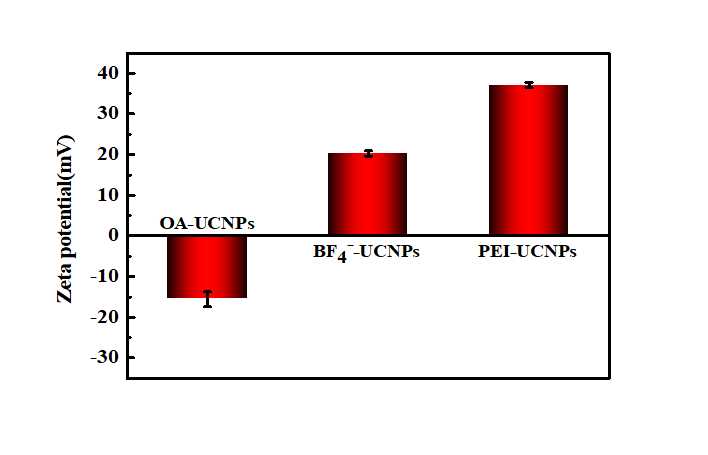


**Supplementary Figure 2.** Zeta potentials of OA-UCNPs, BF^4-^-UCNPs, PEI-UCNPs.

# Dynamic light scattering of AuNPs

The dynamic light scattering (DLS) of AuNPs before and after modification of DNAzyme was tested, respectively. As shown in Supplementary Figure S3, the result showed that the average particle size of AuNPs modified with DNAzyme increased significantly, indicating that DNAzyme could be well modified on the surface of AuNPs.


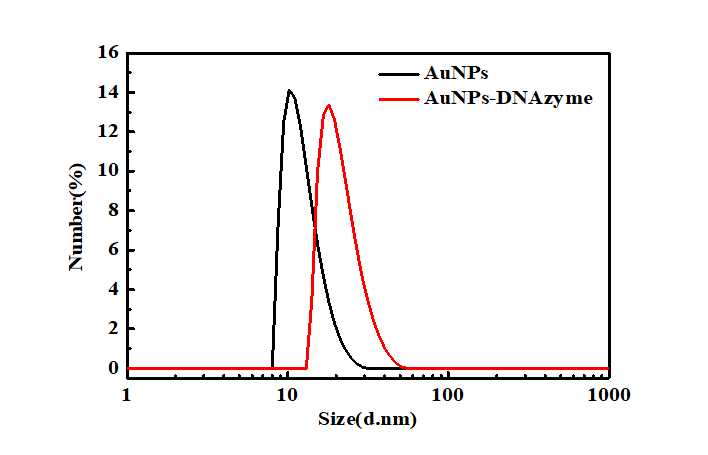


**Supplementary Figure 3.** DLS characterization of AuNPs before and after DNAzyme modification.

# Salt-tolerance of AuNPs-DNAzyme

0.2 M sodium chloride was added to the reaction solutions of AuNPs-DNAzyme and AuNPs, respectively. The color changes were observed after the two groups of solutions were vortexed for 5 min. As shown in Supplementary Figure S4, the results showed that the color of the AuNPs-DNAzyme reaction solution remained red, and the color of the AuNPs reaction solution changed to blue, indicating that DNAzyme was successfully modified on the surface of AuNPs, which could prevent the aggregation of AuPNs (Park et al., 2018; Wang et al., 2021).

**
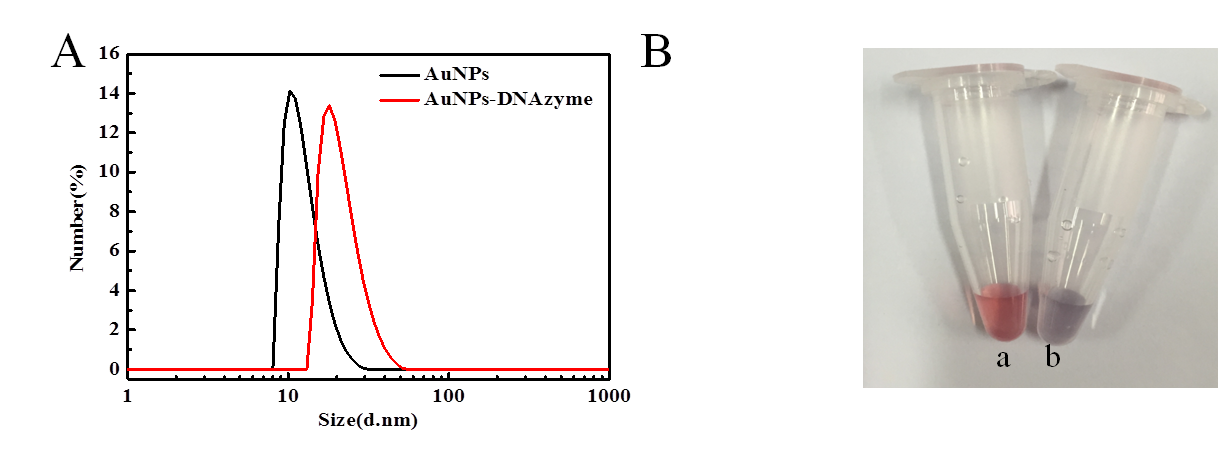
**

**Supplementary Figure 4.** Comparison of salt-tolerance test: AuNPs-DNAzyme solution after adding NaCl (a), AuNPs solution after adding NaCl (b).

# Optimized processing of filter paper

The filter paper and oxidized filter paper both immobilized with PEI-UCNPs were treated by ultrasonic and immersion, respectively, and then washed for different times to observe the changes of the UCL intensity. As shown in Supplementary Figure S5A, after ultrasonic treatment, the UCL intensity on the surface of filter paper and oxidized filter paper gradually weakened with the increase of washing times. Furthermore, the UCL intensity on filter paper attenuated more significantly than the UCL intensity on oxidized filter paper. As shown in Supplementary Figure S5B, after soaking treatment, the loss of the UCL intensity on the surface of the filter paper is more obvious, and the UCL intensity on the surface of oxidized paper remained almost unchanged. It shows that the oxidized filter paper has more pore structure (Yao et al., 2017), which is more stable for the fixation of PEI-UCNPs, and soaking is more suitable for the treatment of PEI-UCNPs after fixation.


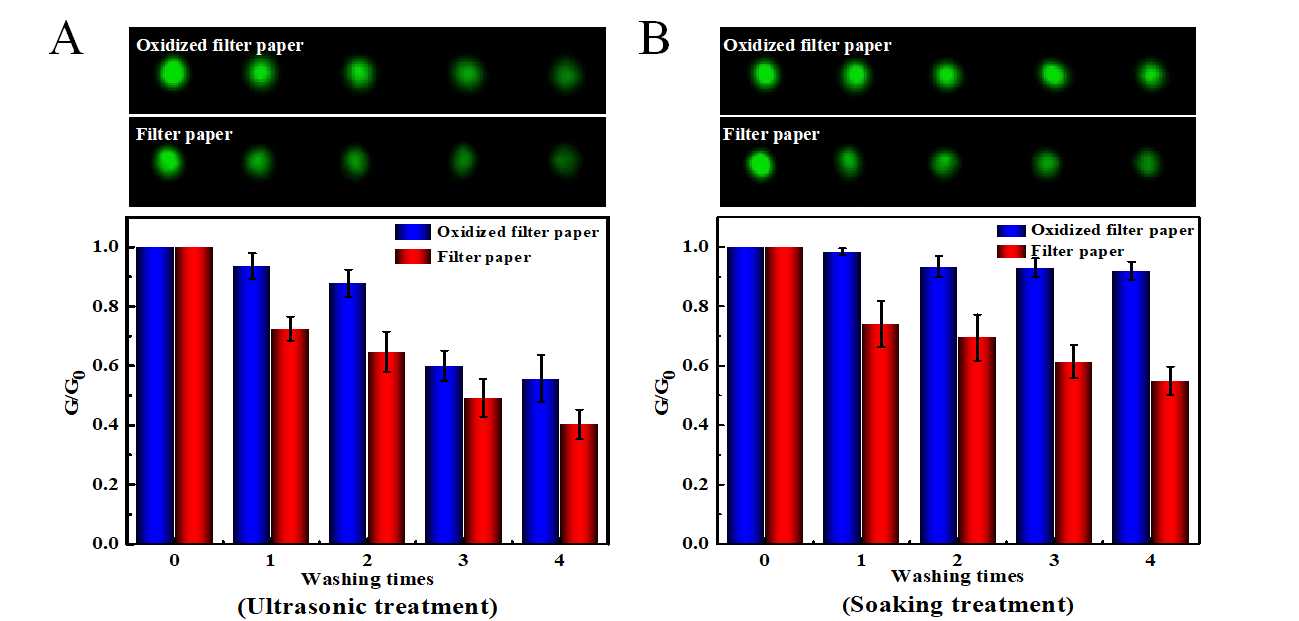


**Supplementary Figure 5.** (A) The observed UCL intensity changes after ultrasonic treatment of PEI-UCNPs immobilized on filter paper and oxidized filter paper, respectively. (B) The observed UCL intensity changes after soaking treatment of PEI-UCNPs immobilized on filter paper and oxidized filter paper, respectively.

# Stability of PEI-UCNPs on oxidized filter paper

The PEI-UCNPs were immobilized on the interface of oxidized filter paper, and the intensity changes of RGB signals were observed within one week. As shown in Supplementary Figure S6, the results showed that the intensity of UCL remained unchanged within a week, indicating that the PEI-UCNPs had good stability on the oxidized filter paper.


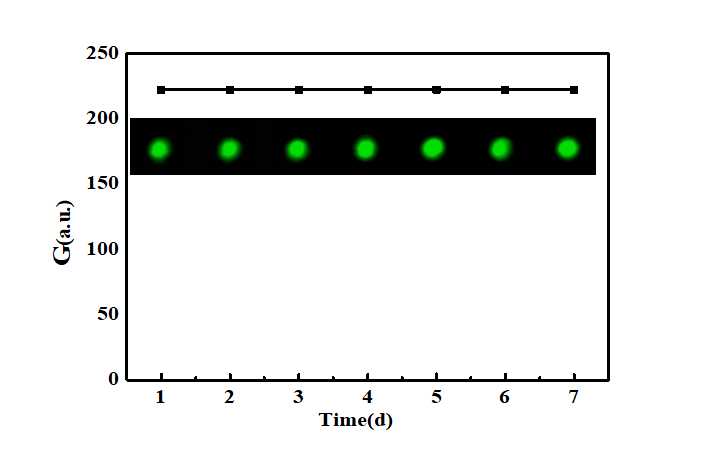


**Supplementary Figure 6.** The changes of UCL intensity of PEI-UCNPs within one week of after immobilization on the interface of oxidized filter paper.

# Quenching of UCL by AuNPs-DNAzyme

Without adding rare-earth ions, the modification concentration of AuNPs-DNAzyme was gradually increased to observe the quenching of UCL. As shown in Supplementary Supplementary Figure S7, the results showed that during the concentration range of 0.0-8.0 nM AuNPs-DNAzyme, the UCL of UCNPs was not significantly quenched, indicating that the design of the sensing mechanism was feasible.


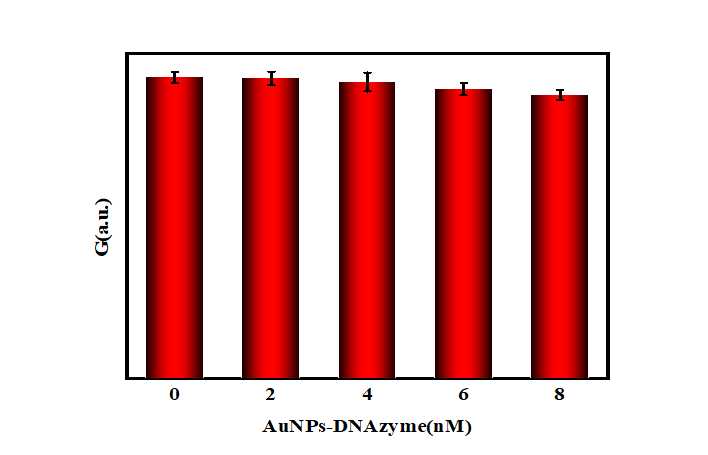


**Supplementary Figure 7.** Quenching of UCL by different concentrations of AuNPs-DNAzyme (0.0, 2.0, 4.0, 6.0 8.0 nM) immobilized on paper (without adding rare-earth ions).

# Optimization of AuNPs-DNAzyme concentration

The concentration of rare-earth ions was fixed at 10 μM and the modification concentration of AuNPs-DNAzyme was gradually increased to observe the quenching of UCL by AuNPs-DNAzyme. As shown in Supplementary Figure S8, The results showed that as the concentration of AuNPs-DNAzyme increased to 6 nM, the quenching of the UCL reached saturated state, indicating that the optimal concentration of AuNPs-DNAzyme was about 6 nM.


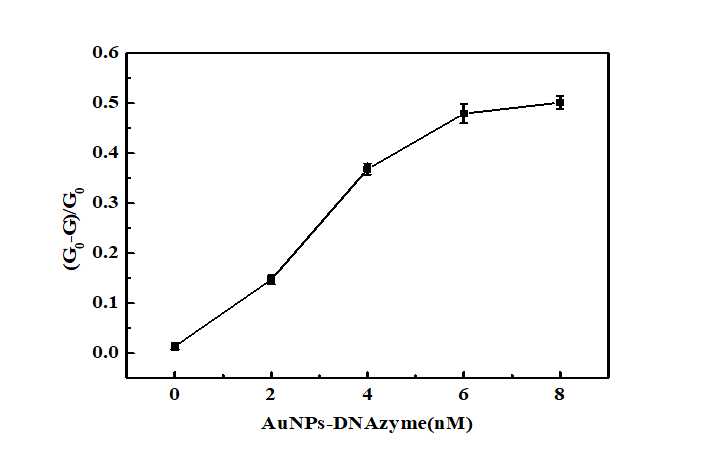


**Supplementary Figure 8.** Quenching of UCL varied with different concentrations of AuNPs-DNAzyme (0.0, 2.0, 4.0, 6.0 8.0 nM), when the concentration of rare-earth ions was 10 μM.

# REFERENCE

Borggrafe, J., Victor, J., Rosenbach, H., Viegas, A., Gertzen, C. G. W., Wuebben, C., et al. (2022). Time-resolved structural analysis of an RNA-cleaving DNA catalyst. Nature 601(7891), 144-149. doi: 10.1038/s41586-021-04225-4.

Lee, K. X., Shameli, K., Yew, Y. P., Teow, S. Y., Jahangirian, H., Rafiee-Moghaddam, R., et al. (2020). Recent Developments in the Facile Bio-Synthesis of Gold Nanoparticles (AuNPs) and Their Biomedical Applications. International Journal of Nanomedicine 15, 275-300. doi: 10.2147/ijn.S233789.

Liu, D. M., Zhao, D., Zhang, D. S., Zheng, K. Z., and Qin, W. P. (2011). Synthesis and Characterization of Upconverting NaYF_4_:Er^3+^, Yb^3+^ Nanocrystals via Thermal Decomposition of Stearate Precursor. Journal of Nanoscience and Nanotechnology 11(11), 9770-9773. doi: 10.1166/jnn.2011.5257.

Park, C., Song, Y., Jang, K., Choi, C. H., and Na, S. (2018). Target switching catalytic hairpin assembly and gold nanoparticle colorimetric for EGFR mutant detection. Sensors and Actuators B-Chemical 261, 497-504. doi: 10.1016/j.snb.2018.01.183.

Wang, L., Liu, Z. J., Cao, H. X., and Liang, G. X. (2021). Ultrasensitive colorimetric miRNA detection based on magnetic 3D DNA walker and unmodified AuNPs. Sensors and Actuators B-Chemical 337, 7. doi: 10.1016/j.snb.2021.129813.

Yao, B., Zhang, J., Kou, T. Y., Song, Y., Liu, T. Y., and Li, Y. (2017). Paper-Based Electrodes for Flexible Energy Storage Devices. Advanced Science 4(7), 32. doi: 10.1002/advs.201700107.
